# Evaluation of the relationship between cardiac remodeling and serum FABP3 in hypertensive patients

**DOI:** 10.1186/s12872-026-06439-8

**Published:** 2026-08-08

**Authors:** Meltem Uyaner Kan, Hasan Kan, Sefa Tatar

**Affiliations:** 1 Department of Medical Biochemistry, Niğde Omer Halisdemir University Training and Research Hospital, Niğde, Türkiye; 2 Department of Cardiology, Niğde Omer Halisdemir University Training and Research Hospital, Niğde, Türkiye; 3 Department of Cardiology, Necmettin Erbakan University, Konya, Türkiye

**Keywords:** Hypertension, Ventricular remodelling, Left ventricular hypertrophy, Fatty acid binding protein 3, H-FABP

## Abstract

**Background:**

Hypertension is a leading cause of cardiovascular disease and all-cause mortality. In addition, owing to the pathological processes it causes in the vascular bed, it leads to end-organ damage, thereby contributing to morbidity. FABP3 (fatty acid binding protein 3) is a protein primarily expressed in the cardiac muscle, skeletal muscle, and kidney, and plays an important role in cardiomyocyte homeostasis.

**Methods:**

Echocardiographic evaluation was performed in 41 normotensive controls and 120 patients with hypertension. Patients with cardiac remodelling (*n* = 50) and hypertrophy (*n* = 33) were identified by calculating the left ventricular mass and relative wall thickness. FABP3 levels were measured in serum samples using ELISA.

**Results:**

FABP3 levels demonstrated an increasing trend (*p* < 0.0001) across the control, non-remodelled hypertensive, remodelling, and hypertrophy groups (1.27 ± 0.81, 1.85 ± 0.75, 2.31 ± 1.06, and 2.85 ± 1.71 ng/ml, respectively). FABP3 levels were significantly but weakly correlated with posterior wall thickness (ρ = 0.29), relative wall thickness (ρ = 0.23), and troponin levels (ρ = 0.2).

**Conclusion:**

These findings suggest that FABP3 may be associated with hypertension-mediated cardiac remodelling and left ventricular hypertrophy. FABP3 may have potential as a supportive biomarker reflecting subclinical cardiac involvement in patients with hypertension.

## Background

The fatty acid-binding protein (FABP) family consists of 14–15 kDa proteins that play a role in the uptake, intracellular metabolism, and transport of long-chain fatty acids [1]. FABPs also play crucial roles in regulating cellular growth and proliferation [2].

Cardiovascular diseases (CVD) significantly contribute to mortality rates worldwide. Atherosclerosis, endothelial dysfunction, and dyslipidemia are important factors in the development of CVD. The role of FABPs in lipid processing in adipocytes, macrophages, and endothelial cells indicates their involvement in CVD pathogenesis [3].

FABP3 (fatty acid binding protein 3, heart type FABP (H-FABP)) is primarily expressed in cardiac muscle and plays an important role in cellular signaling, cardiomyocyte homeostasis, and lipid transport. Owing to the amphipathic structure of fatty acids, their accumulation and storage in membranes can disrupt their structure and function. Decreased FABP3 levels in ischemic or inflammatory processes may lead to the progression of cardiac damage following myocyte dyshomeostasis. FABP3 is an important biomarker in the pathophysiological process of myocardial dysfunction following cellular damage [4].

Effective management of hypertension is a key objective in reducing cardiovascular morbidity and mortality. Elevated blood pressure can be effectively and tolerably managed through lifestyle modification and pharmacological interventions. However, the global rate of blood pressure control remains suboptimal [5]. Hypertension remains the leading modifiable determinant of overall and cardiovascular morbidity and mortality globally [6].

Hypertension-mediated organ damage (HMOD) refers to structural or functional changes in the major and minor arteries or end organs (brain, heart, kidneys, and eyes) caused by high blood pressure [7]. With the increased utilization of transthoracic echocardiography (TTE) for imaging, HMOD is more frequently identified in asymptomatic patients [8].

HMOD is a strong indicator of comprehensive cardiovascular risk [5]. Early detection of structural and functional cardiac alterations related to hypertension is important for preventing cardiovascular events. This study aimed to investigate the association between FABP3 levels and left ventricular hypertrophy and to assess the potential clinical relevance of FABP3 in hypertensive cardiac remodelling.

## Patients and methods

**Fig. 1:**
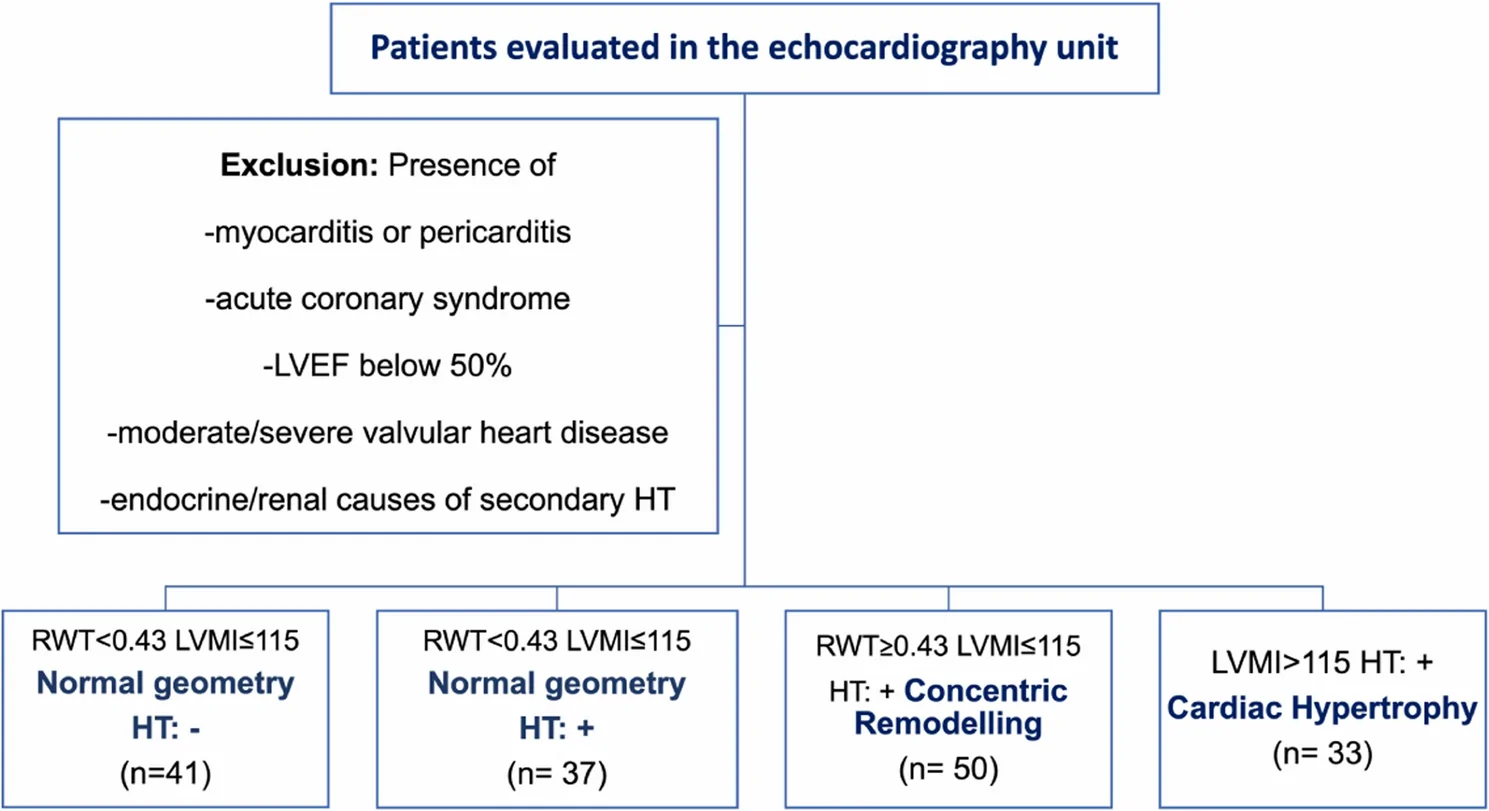
Flowchart of patient selection and distribution of patients into study groups

Participants were individuals with hypertension who attended the Echocardiography Unit at the Faculty of Medicine, Necmettin Erbakan University. To minimize sex-related confounding factors, and because the diagnostic criteria for left ventricular hypertrophy and remodelling vary between sexes, only male patients were included. A total of 120 hypertensive patients and 41 normotensive controls were included in our cross-sectional study, and an average Home BP measurement of ≥ 135/85 mmHg was used to diagnose hypertension [6]. Although the control group was normotensive, a small proportion of participants were receiving beta-blockers or ACE inhibitors/ARBs for indications unrelated to hypertension, such as arrhythmia, migraine prophylaxis, or other non-hypertensive clinical conditions. None fulfilled the diagnostic criteria for hypertension. The study protocol was approved by the Ethics Committee of Necmettin Erbakan University (approval no. 2024/5119), and written informed consent was obtained from all participants. The exclusion criteria for the study and the allocation of patients to the study groups are shown in Fig. 1.

TTE was performed in all groups who participated in the study in the left lateral decubitus position using the Epiq 7 ultrasound system (Philips Medical Systems, Andover, MA, USA) with a 5 − 1 MHz transducer. Echocardiographic measurements were performed by two different cardiologists, both experts who were unaware of the patients’ clinical and demographic characteristics. Echocardiographic measurements were performed in accordance with the guidelines of the American Society of Echocardiography (ASE) and the European Association of Cardiovascular Imaging (EACVI) [9]. Left ventricular geometry and diastolic and systolic dysfunction were evaluated using TTE.

Left ventricular geometry was evaluated by calculating relative wall thickness (RWT) using the following formula:

* RWT=2PWTd/LVIDd*

where PWTd: Posterior Wall Thickness at end-diastole and LVIDd: Left Ventricular Internal Diameter at end-diastole.

Left ventricular mass (LVM) has been calculated using the formula:

$$\:LVM=0.8\left\{1.04\left[{\left(IVSd+LVIDd+PWTd\right)}^{3}-{\left(LVIDd\right)}^{3}\right]\right\}+0.6g$$

where IVSd: Interventricular Septal thickness at end-diastole, LVIDd: Left Ventricular Internal Diameter at end-diastole, and PWTd: Posterior Wall Thickness at end-diastole.

The Body Surface Area (BSA) was calculated using the Mosteller formula, with height measured in cm and weight measured in kg:

$$\:BSA=\sqrt{height\times\:weight/3600}$$

The left ventricular mass index (LVMI) was calculated using the following formula [10]:

*LVMI=LVM/BSA*

The left ventricular concentric geometry criterion for HMOD, in accordance with the guidelines, was defined as RWT ≥ 0.43 [6]. Patients with RWT ≥ 0.43 and LVMI ≤ 115 were considered to have concentric remodelling, and those with LVMI > 115 were classified into the cardiac hypertrophy group. The groups were as follows: normotensive control with normal left ventricular geometry (*n* = 41), hypertensive (HT) with normal geometry (*n* = 37), cardiac remodelling (*n* = 50), and cardiac hypertrophy (*n* = 33).

Serum samples were obtained from patients in a fasting state, subjected to centrifugation at 1500 × g for 15 min, and subsequently stored at − 80 °C until analysis. FABP3 levels were measured using a Bioassay Technology Laboratory Human FABP3 ELISA Kit (Lot No: 202503020, Cat. No: E4045Hu, Shanghai, China) according to the manufacturer’s instructions. The sensitivity of the ELISA kit was 0.01 ng/ml; the intra-assay coefficient of variation was below 8%, and the inter-assay coefficient of variation was below 10%. To minimize batch effects in the measurements, the samples were processed in a single session, and internal quality control samples were used during analyses.

### Statistical analyses

IBM SPSS Statistics version 26.0 (IBM Corp., Armonk, NY, USA) and GraphPad Prism version 9.2 (GraphPad Software, San Diego, CA, USA) were used for the statistical analyses. Descriptive statistical analysis was conducted using frequency, percentage, mean, and standard deviation. Normality was assessed using the Shapiro–Wilk test. In four independent groups, the data were evaluated using ANOVA or the Kruskal-Wallis test, as appropriate according to the distribution of the data. Post hoc analyses were adjusted using the Benjamini–Krieger–Yekutieli false discovery rate procedure. The association between FABP3 concentration and other quantitative variables was assessed using Spearman’s rank correlation analysis. Initially, univariate linear regression analyses were conducted to assess FABP3 levels. Subsequently, to account for the influence of the group and potential confounding variables, a multivariable linear regression model was developed, incorporating both the group and calcium channel blocker use. To evaluate the effect modification, the interaction term group × CCB was incorporated into the model, and its significance was assessed. Statistical significance was set at *p* < 0.05.

## Results

Table 1 provides a comprehensive overview of the demographic characteristics, smoking and medication use, biochemical measurements, echocardiographic parameters, and FABP3 levels of the study groups.

**Table 1:** Baseline characteristics of the study groups

Characteristic	Normotensive Group	Hypertensive Group	Cardiac Remodeling Group	Cardiac Hypertrophy Group	*P*- value
	*n* = 41 (25%)	*n* = 37 (23%)	*n* = 50 (31%)	*n* = 33 (20%)	
Age (years)	58.4 ± 10.8	61.3 ± 9.1	63.9 ± 8.6	63.1 ± 11.3	0.071
Smoke (%)	44%	23%	34%	46%	0.32
BMI (kg/m^2^)	29.5 (25.4, 32.9)	30.1 (26.0, 31.7)	28.1 (25.6, 30.9)	26.7 (25.2, 29.1)	0.19
BSA (m^2^)	1.99 (1.80, 2.10)	1.96 (1.89, 2.10)	1.92 (1.82, 2.06)	1.88 (1.71, 1.96)	0.094
Beta-blocker usage (%)	19.50%	73.90%	62.10%	76.90%	< 0.001
ACEi/ARB usage (%)	4.90%	65.20%	75.90%	76.90%	< 0.001
CCB usage (%)	0%	39.10%	27.60%	38.50%	0.021
Diuretic usage (%)	7.30%	26.10%	31%	38.50%	0.031
Statin usage (%)	36.80%	52.20%	55.20%	46.20%	0.637
Antidiabetic drug usage (%)	21.10%	52.20%	34.50%	30.80%	0.199
Antiaggregant drug usage (%)	68.40%	60.90%	65.50%	61.50%	0.956
Anticoagulant drug usage (%)	5.30%	21.70%	3.40%	15.40%	0.144
LVIDd (cm)	4.80 (4.70, 5.00)	4.90 (4.70, 5.10)	4.30 (4.20, 4.60)	5.30 (4.60, 5.60)	< 0.001
IVSd (cm)	1 (0.9–1.1)	1 (1-1.1)	1.1 (1.1–1.2)	1.2 (1.1–1.3)	< 0.001
PWTd: (cm)	1 (0.9-1)	1 (0.9-1)	1.1 (1-1.1)	1.1 (1-1.2)	< 0.001
LVM (g)	182 (153, 192)	182 (153, 194)	166 (152, 194)	242 (227, 262)	< 0.001
LVMI (g/ m^2^)	91 (75, 98)	88 (76, 98)	90 (79, 102)	131 (121, 153)	< 0.001
RWT	0.40 (0.38, 0.42)	0.40 (0.38, 0.41)	0.48 (0.46, 0.51)	0.42 (0.37, 0.49)	< 0.001
Urea (mg/dl)	29 (23, 34)	32 (26, 36)	32 (26, 39)	35 (28, 41)	0.14
Creatinine (mg/dl)	0.94 (0.83, 1.03)	0.91 (0.82, 1.00)	0.97 (0.87, 1.06)	0.92 (0.83, 1.10)	0.46
CRP (mg/l)	2 (1, 5)	2 (1, 4)	2 (1, 5)	4 (1, 11)	0.37
LDL-C (mg/dl)	100 (74, 127)	87 (52, 120)	78 (66, 111)	94 (74, 112)	0.33
hs-cTnT(ng/l)	5 (4, 7)	7 (4, 11)	9 (5, 12)	10 (6, 21)	0.004
FABP3 (ng/ml)	1.27 ± 0.81	1.85 ± 0.75	2.31 ± 1.06	2.85 ± 1.71	< 0.0001

Continuous variables are summarized as median (IQR) or mean ± standard deviation (SD). For comparisons between groups, ANOVA or Kruskal–Wallis rank sum tests were used, as appropriate, for the distribution. Categorical variables are presented as n (%) and were analyzed using Pearson’s chi-squared test. False discovery rate correction for multiple tests was implemented. The groups were defined based on RWT ≥ 0.43 and LVMI > 115 g/m²

*Abbreviations*: *BMI* Body Mass Index, *BSA* Body Surface Area (m²), *ACEi/ARB* ACE inhibitor/angiotensin receptor blocker, *CCB* calcium channel blocker, *LVIDd* left ventricular internal diameter in diastole, *IVSd* interventricular septal thickness in diastole, *PWTd* posterior wall thickness in diastole, *LVM* left ventricular mass, *LVMI* left ventricular mass index, *RWT* relative wall thickness, *CRP* C-reactive protein, *LDL-C* low-density lipoprotein cholesterol, *hs-cTnT* high-sensitivity cardiac troponin T, *FABP3* fatty acid-binding Protein 3

**Fig. 2:**
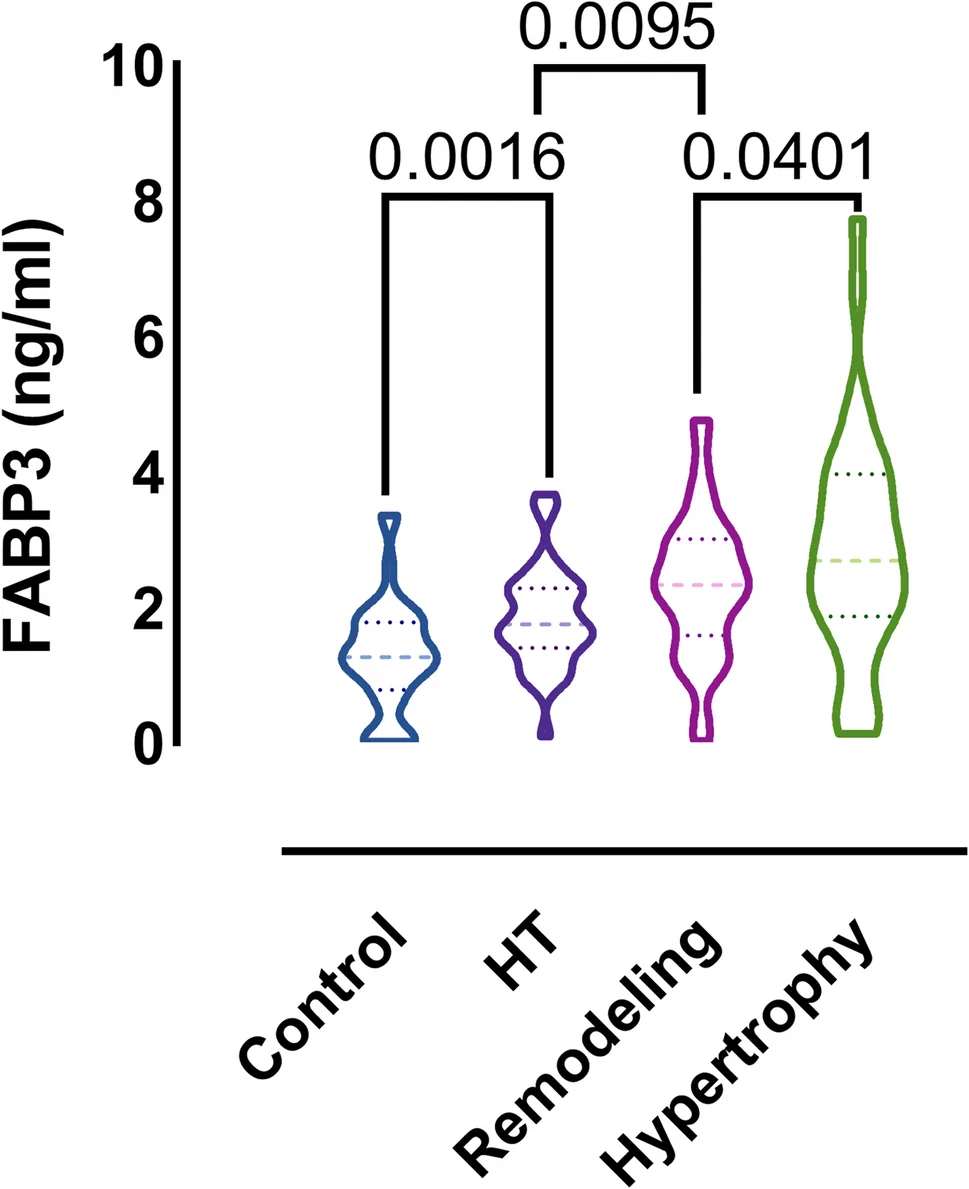
Distribution of serum FABP3 levels across the normotensive control (*n* = 41), hypertensive with normal left ventricular geometry (*n* = 37), cardiac remodelling (*n* = 50), and cardiac hypertrophy (*n* = 33) groups. The width of each violin represents the estimated density of the observations. The central dashed lines indicate the medians, whereas the lower and upper dotted lines represent the 25th and 75th percentiles, respectively. The 25th percentile, median, and 75th percentile values were 0.77, 1.26, and 1.77 ng/mL in the normotensive control group; 1.40, 1.75, and 2.28 ng/mL in the hypertensive group with normal left ventricular geometry; 1.58, 2.33, and 3.00 ng/mL in the cardiac remodelling group; and 1.86, 2.68, and 3.96 ng/mL in the cardiac hypertrophy group, respectively. Mean ± standard deviation values were 1.27 ± 0.81, 1.85 ± 0.75, 2.31 ± 1.06, and 2.85 ± 1.71 ng/mL, respectively. Pairwise post hoc p values were adjusted using the two-stage linear step-up procedure of Benjamini, Krieger, and Yekutieli

FABP3 levels demonstrated an increasing trend (*p* < 0.0001) across the normotensive (*n* = 41), non-remodelled hypertensive (*n* = 37), remodelling (*n* = 50), and hypertrophy (*n* = 33) groups (1.27 ± 0.81, 1.85 ± 0.75, 2.31 ± 1.06, 2.85 ± 1.71 ng/ml, respectively). In the post hoc analysis, significant statistical differences were observed between the normotensive and non-remodelled hypertensive groups (*p* = 0.0016), between the non-remodelled hypertensive and remodelling groups (*p* = 0.0095), and between the remodeling and hypertrophy groups (*p* = 0.0401) (Fig. 2).

The correlation between patient parameters and FABP3 levels was evaluated using Spearman’s correlation test. FABP3 levels showed a statistically significant but weak correlation with posterior wall thickness (ρ = 0.29), relative wall thickness (ρ = 0.23), and troponin levels (ρ = 0.2) (Table 2).

**Table 2:** Correlation of FABP3 with cardiac and biochemical parameters

	Spearman ρ	%95 CI	*p* value
PWTd	0.29	0.14–0.43	0.0002
RWT	0.23	0.078–0.38	0.0028
hs-cTnT	0.2	0.03–0.36	0.0181
LVM	0.19	0.031–0.34	0.0162
LVMI	0.17	0.012–0.32	0.0301
IVSd	0.17	0.013–0.32	0.0293

*Abbreviations*: *PWTd* Posterior wall thickness at end-diastole, *RWT* Relative wall thickness, *hs-cTnT* high-sensitivity cardiac troponin T, *LVM* Left ventricular mass, *LVMI* Left ventricular mass index, *IVSd* interventricular septal diameter, *%95 CI* %95 Confidence Interval

In the univariate regression analysis, all variables were assessed, revealing a significant association between CCB use and FABP3 levels (*p* = 0.009). The multivariate model indicated that the differences in FABP3 levels between the groups were independent of CCB use (Adjusted R^2^ =0.214) (Table 3). The interaction effect between the group and CCB (Group × CCB) was not significant (*p* = 0.255). Interaction analysis revealed that CCB use did not alter the association between the study groups and FABP3 levels. Thus, the observed FABP3 elevation appears to be primarily associated with cardiac geometric changes rather than CCB use.

**Table 3:** Multivariable linear regression analysis of FABP3 levels

Predictor	Coefficient (B)	SE	95% CI	t	*p* value
Intercept	0.59	0.31	–0.02, 1.20	1.92	0.058
CCB usage	0.52	0.27	–0.03, 1.06	1.9	0.062
Patient group	0.48	0.12	0.24, 0.72	4.02	< 0.001

*Abbreviations*: *SE* standard error, *95% CI* 95% confidence interval, *p* significance level; *CCB* Calcium Channel Blocker. The group × CCB interaction was tested and found to be non-significant (*p* = 0.255)

## Discussion

In our study, FABP3 levels increased significantly across groups representing progressively greater hypertension-mediated cardiac structural involvement. The mean FABP3 levels in the normotensive, hypertension, cardiac remodelling, and cardiac hypertrophy groups were 1.27 ± 0.81; 1.85 ± 0.75; 2.31 ± 1.06; and 2.85 ± 1.71 ng/ml, respectively. This increase suggests a progressive association between hypertensive cardiac alterations and FABP3 levels. FABP3 levels showed a statistically significant but weak correlation with PWTd, RWT, and hs-cTnT levels. FABP3 is a small cytosolic protein that can be rapidly released into the bloodstream from myocardial cells, and FABP3 levels may be associated with pressure overload due to hypertension and accompanying myocardial stress, injury, and metabolic dysfunction. The elevation of FABP3 levels in individuals with hypertension suggests that this protein may reflect myocardial stress associated with hypertension-mediated structural cardiac changes. Additionally, it may serve as a biochemical indicator of increased left ventricular mass and cardiac remodelling, However, given the modest correlation coefficients observed in the present study, FABP3 should not be considered a stand-alone marker for the diagnosis of cardiac remodelling or left ventricular hypertrophy. Rather, it may have potential as an adjunctive biomarker when interpreted together with echocardiographic findings, clinical characteristics, and established cardiac biomarkers. Prospective longitudinal studies are required to determine whether FABP3 provides incremental diagnostic and prognostic information beyond current echocardiographic assessments and established cardiac biomarkers, clarify its potential value in long-term cardiovascular risk stratification, and establish clinically relevant threshold values before it can be considered for clinical application.

Numerous biomarkers have been proposed for cardiac remodelling associated with hypertension. According to current hypertension guidelines, an hs-cTnT level above the 99th-percentile upper reference limit is one of the criteria used to define cardiac HMOD [6]. In our study, the correlation between FABP3 and troponin levels suggests that both proteins are associated with hypertensive myocardial stress.

NT-proBNP is an established biomarker of myocardial stretch and haemodynamic wall stress, whereas FABP3 may more directly reflect cardiomyocyte injury, cellular stress, and disturbances in myocardial lipid metabolism. Therefore, these biomarkers may provide complementary rather than interchangeable information. However, NT-proBNP was not measured in the present study, and the relative or incremental value of FABP3 could not be determined. Future studies directly comparing FABP3 with NT-proBNP are required to establish whether FABP3 adds diagnostic or prognostic value beyond established cardiac biomarkers.

A study conducted on 249 patients with stable angina pectoris showed that FABP3 was significantly associated with LVMI and ejection fraction [11]. These results indicate that FABP3 expression is correlated with structural and functional changes, even in the nonhypertensive group.

Although studies specific to hypertension and FABP3 are limited, studies conducted in the general population have identified a link between FABP3, cardiovascular risk factors, and mortality. In a comprehensive cohort study, FABP3 concentration was positively correlated with cardiovascular risk factors, including hypertension, diabetes mellitus, and obesity. Individuals in the highest quartile of FABP3 exhibited an increased risk of both total and cardiovascular mortality [12]. FABP3 has been proposed as a biomarker for ischemic conditions such as coronary heart disease. Compared to troponin, FABP3 exhibits an earlier increase in blood levels, occurring approximately 30 min after the onset of myocardial ischemia [3].

Experimental studies have further substantiated the link between FABP3 and hypertrophy. In transverse aortic constriction (TAC) models, cardiac hypertrophy stimuli increased FABP3 expression; it has been shown that ablation of the FABP3 gene leads to an aggravation of hypertrophy and dysfunction induced by TAC. Multi-omic analysis has demonstrated metabolic disorders, such as increased glycolysis, decreased fatty acid oxidation, and impaired ATP production, in FABP3 ablation. This imbalance in energy metabolism, coupled with metabolic stress and impaired FA trafficking, may contribute to cardiac remodelling. It has been found that FABP3 maintains metabolic balance by interacting with PPARα, and that the adverse effects can be partially reversed with the PPARα agonist fenofibrate [13]. Research has indicated that circulating FABP3 levels are elevated in the presence of hypertrophy in both mice and humans [14]. These findings suggest that FABP3 plays a role in myocardial metabolism and may respond to hypertrophic stimuli.

Studies conducted on other pathologies also support the relationship between FABP3 and cardiac remodelling. A significant correlation was identified between FABP3 levels and left ventricular (LV) dimensions in individuals with aortic valve disease and concurrent hypertension whose troponin T levels remained below the normal range [15].

Advanced left ventricular dilatation and systolic dysfunction may be associated with ongoing cardiomyocyte injury and metabolic stress. A previous study reported the prognostic value of serum FABP3 in patients with non-ischaemic dilated cardiomyopathy [16]. However, because patients with an LVEF below 50% were excluded from the present study, it remains unknown whether FABP3 levels continue to increase in patients who develop advanced ventricular dilatation and systolic dysfunction. Longitudinal studies extending beyond the remodelling stages evaluated in the present study to include advanced ventricular dilatation and overt systolic dysfunction are needed to clarify this relationship.

At the molecular level the regulation of FABP3 through CpG methylation has been associated with insulin resistance, lipid profile disorders, and cardiovascular phenotypes related to the metabolic syndrome [17]. FABP3 may play a role in cardiac injury through its participation in lipid metabolism.

It has also been shown that FABP3 overexpression triggers ER stress by altering the lipid composition of the cell membrane. In one study, elevated FABP3 levels modified the membrane lipids of young muscle cells to resemble those found in aged muscle tissue. This alteration results in a reduction in polyunsaturated phospholipid acyl chains within the membrane and an increase in sphingomyelin and lysophosphatidylcholine levels. This lipid remodelling induces ER stress via the PERK-eIF2α pathway [18]. A similar process may occur in cardiomyocytes; chronic ER stress can lead to dysregulation of protein synthesis, cell damage, apoptosis, and fibrosis, thereby laying the groundwork for cardiac remodelling.

Studies have demonstrated that elevated FABP3 levels affect intracellular signaling pathways. It has been reported that in myocardial ischemia, the overexpression of FABP3 increases the phosphorylation of MAP kinases, decreases the level of phospho-Akt, and enhances apoptosis and remodelling after infarction. The same study demonstrated that FABP3 deficiency had protective effects against ischemic heart injury by reducing cardiomyocyte apoptosis and limiting cardiac remodelling after MI [19].

In a cell study conducted under oxidative stress, excess FABP3 led to increased oxidative stress markers, measured by elevated levels of pro-inflammatory cytokines such as IL-6, IL-1β, and TNF-α, as well as indicators of oxidative damage, including higher malondialdehyde levels and reduced superoxide dismutase and glutathione levels. At the same time these effects were reversed by FABP3 knockdown [20]. These findings suggest that FABP3 triggers inflammatory pathways via lipid peroxidation. Similarly, increased lipid peroxidation and oxidative stress in cardiomyocytes can accelerate loss of function by contributing to cell damage and interstitial fibrosis.

Elevated FABP3 blood levels may also result from the release of FABP3 during cardiac injury. FABP3 levels increase in conditions such as myocardial infarction [21]. Chronic ischemia, increased oxidative stress, and myocyte apoptosis can also induce the release of FABP3 from cardiac muscle tissue. Therefore, elevated serum FABP3 levels may reflect myocardial injury and greater hypertension-mediated cardiac structural involvement.

Although FABP3 is commonly referred to as heart-type fatty acid-binding protein, it is not entirely cardiac-specific. FABP3 is also expressed in skeletal muscle, and its circulating concentration may be influenced by skeletal muscle injury [22] or recent strenuous physical activity. In addition, reduced renal clearance may contribute to elevated circulating FABP3 levels [23]. In the present study, FABP3 was not significantly correlated with serum creatinine, urea, or eGFR (all *p* > 0.05), and serum creatinine and urea levels were comparable among the study groups. These findings suggest that overt variations in renal function were unlikely to account for the progressive increase in FABP3 levels across the study groups. Nevertheless, routinely assessed renal indices may not fully capture subtle alterations in renal function or FABP3 clearance. Moreover, although no participant had a known skeletal muscle disease, serum creatine kinase levels and recent physical activity were not systematically assessed. Therefore, potential contributions from subclinical skeletal muscle injury, exercise-related FABP3 release, or subtle alterations in renal clearance cannot be completely excluded. These extracardiac determinants may limit the cardiac specificity of FABP3 and support its interpretation as a potential adjunctive biomarker rather than a stand-alone marker of hypertension-mediated cardiac involvement.

### Limitations

This study has several limitations that should be considered when interpreting the findings. In particular, the study population was relatively small and recruited from a single center, which may limit the generalizability of the results. The exclusive focus on male participants in our study restricted the extent to which the findings can be generalized to the entire population. Recent hypertension guidelines have highlighted significant gaps in sex-specific evidence regarding the epidemiology, risk determinants, hormonal and genetic mechanisms, and cardiovascular risk attribution in patients with hypertension, emphasizing the need for prospective, sex-stratified research [6]. Our study focused exclusively on male patients to ensure a more homogeneous cohort and minimize potential confounding factors arising from sex-related physiological and pathophysiological variability. Although FABP3 was not significantly correlated with renal parameters, subtle renal effects, subclinical skeletal muscle injury, and recent physical activity could not be completely excluded. Moreover, the clinical utility of FABP3 requires the establishment of validated diagnostic cut-off values and reference ranges. In addition, the cross-sectional design precludes causal inference. Therefore, large-scale multicenter prospective studies are warranted to evaluate the diagnostic utility of FABP3 in hypertensive remodelling and hypertrophy, define clinically relevant diagnostic thresholds, and assess their reproducibility across diverse populations.

It is essential to conduct further studies to understand FABP3’s involvement in organ damage due to hypertension. Therefore, it is necessary to elucidate the pathophysiological mechanisms of FABP3. Although hypertension-mediated organ damage predominantly affects left ventricular geometry, right ventricular remodeling may also occur secondary to ventricular interdependence, pulmonary vascular changes, and chronic pressure overload. Because comprehensive right ventricular echocardiographic parameters (e.g., TAPSE, RV fractional area change, or RV strain) were not systematically evaluated in the present study, the relationship between FABP3 and right ventricular structure or function could not be assessed. Future studies should examine the prognostic role of FABP3 and evaluate its relationship with right ventricular structure and function using comprehensive echocardiographic parameters. The levels and variations in FABP3 can also be assessed using alternative biological samples, such as urine. Urinary FABP3 levels have been shown to be associated with peripheral arterial disease [24] and early diabetic kidney disease [25]. Similarly, it is necessary to study the renal excretion and urinary levels of FABP3 in hypertensive nephropathy. BNP and NT-proBNP were not assessed, limiting direct comparison with established biomarkers of cardiac stress. In future research, FABP3 could be assessed alongside or against BNP and troponin levels to determine whether it adds diagnostic or prognostic value. A comparison of these markers may help clarify FABP3’s clinical relevance. If FABP3 is to be used in routine care, clear cutoff values for diagnosis, prognosis, and treatment decisions must be defined.

## Conclusions

In conclusion, FABP3 levels progressively increased across groups with greater hypertension-mediated cardiac structural involvement and were higher than those in the normotensive group. FABP3 showed statistically significant but weak correlations with posterior wall thickness, relative wall thickness, and hs-cTnT levels. These findings suggest that FABP3 may have potential as an adjunctive biomarker of hypertension-mediated cardiac remodelling. Nevertheless, these findings should be interpreted cautiously because FABP3 is not entirely cardiac-specific and may be influenced by extracardiac sources. Future studies should specifically evaluate the clinical utility of FABP3 in supporting early detection, risk stratification, and monitoring of therapeutic responses in patients with hypertension.

## Data Availability

The data can be shared by the corresponding author upon reasonable request.

## Abbreviations

ACEi/ARB: Angiotensin-converting enzyme inhibitor/angiotensin receptor blocker
ASE: American Society of Echocardiography
ATP: Adenosine triphosphate
BMI: Body mass index
BNP: B-type natriuretic peptide
BSA: Body surface area
CCB: Calcium channel blocker
CRP: C-reactive protein
CVD: Cardiovascular disease
EACVI: European Association of Cardiovascular Imaging
ELISA: Enzyme-linked immunosorbent assay
FABP3: Fatty acid-binding protein 3
H-FABP: Heart-type fatty acid-binding protein
HMOD: Hypertension-mediated organ damage
hs-cTnT: High-sensitivity cardiac troponin T
HT: Hypertension
IL-1β: Interleukin-1 beta
IL-6: Interleukin-6
IQR: Interquartile range
IVSd: Interventricular septal thickness at end-diastole
LDL-C: Low-density lipoprotein cholesterol
LVEF: Left ventricular ejection fraction
LVIDd: Left ventricular internal diameter at end-diastole
LVM: Left ventricular mass
LVMI: Left ventricular mass index
PPARα: Peroxisome proliferator-activated receptor alpha
PWTd: Posterior wall thickness at end-diastole
RWT: Relative wall thickness
SE: Standard error
TAC: Transverse aortic constriction
TNF-α: Tumor necrosis factor-alpha
TTE: Transthoracic echocardiography
